# Comparison of inflammatory markers between brucella and non-brucella epididymo-orchitis

**DOI:** 10.1590/S1677-5538.IBJU.2018.0004.0

**Published:** 2018

**Authors:** Ali Cift, Mehmet Ozgur Yucel

**Affiliations:** 1 Department of Urology, Faculty of Medicine, Adiyaman University, Adiyaman, Turkey

**Keywords:** Brucella, Infectious Disease Medicine, Hematologic Neoplasms

## Abstract

**Objectives:**

Brucellosis is a multi-system infectious disease that is associated with inflammation, which causes an increase in acute phase reactants. Hematological inflammatory markers of brucellosis include mean platelet volume (MPV), red cell distribution width (RDW), neutrophil/lymphocyte ratio (NLR), and platelet/lymphocyte ratio (PLR). In this study, we aimed to evaluate the diagnostic value of hematological inflammatory markers in Brucella epididymo-orchitis (BEO), and to investigate the utility of these markers for differential diagnosis from non-Brucella epididymo-orchitis (non-BEO).

**Materials and Methods:**

We retrospectively reviewed the records of 22 BEO and 50 non-BEO patients. Hematological parameters were recorded and compared between the two groups. The main diagnostic criteria for BEO were positive clinical findings (i.e., testicular pain, tenderness and scrotal swelling), a positive Rose Bengal test result, standard tube agglutination (STA) titer ≥ 1/160, and/or a positive blood culture.

**Results:**

The most decisive factors in discriminating between BEO and non-BEO were NLR, RDW, and MPV, in decreasing order of their strength. Regardless of other factors, NLR values < 2.3 significantly increased the odds of BEO (OR=8.080, 95% CI: 1.929-33.843, p=0.004). After adjusting for other factors, RDW values >14.45% significantly increased the odds of BEO (OR=7.020, 95% CI: 1.749-28.176, p=0.006). Independent of the other factors, patients with MPV < 7.65 fL had a 6.336 times higher risk for BEO (95% CI: 1.393 - 28.822, p=0.017).

**Conclusion:**

Hematological inflammatory markers such as NLR, RDW, and MPV can aid in the differential diagnosis of BEO and non-BEO.

## INTRODUCTION

Brucellosis is an endemic zoonotic disease caused by gram-negative coccobacilli that affects more than half a million patients every year (1). Human brucellosis can affect the genitourinary system, the central nervous system, the respiratory system, and the cardiovascular system via hematogenous spread, and symptoms include night sweating, fever, weight loss, joint pain, anorexia, and fatigue (2). Approximately 20-40% of cases have focal complications and single-organ involvement, which can occur in almost every organ system (3). Focal involvement of the urogenital system is seen in 2-10% of patients. Of males affected by brucellosis, 2-20% have epididymo-orchitis, which is the most commonly involved site in the genitourinary system (4). Thus, Brucella epididymo-orchitis (BEO) must be considered in the differential diagnosis of epididymo-orchitis (5). When treated in a timely manner, BEO has a good prognosis, but any delay in its diagnosis and treatment can lead to serious complications that may require orchiectomy, such as testicular abscess (6).

Brucellosis is characterized by an inflammatory state that results in increased acute phase reactants. Hematological inflammatory markers include white blood cell (WBC) count, platelet (PLT) count, mean platelet volume (MPV), platelet distribution width (PDW), red cell distribution width (RDW), neutrophil/lymphocyte ratio (NLR), platelet/lymphocyte ratio (PLR), and monocyte/lymphocyte ratio (MLR) (7).

MPV represents the mean platelet size in the blood. It can be altered in various diseases such as cancer, thrombosis, sepsis, respiratory distress syndrome, and acute appendicitis (8, 9). PDW represents the variation in platelet diameter, while RDW is a measure of the differences in the sizes or the volumes of red cells. WBC count, eosinophil count, and leukocyte ratios (e.g., NLR) can be altered in carcinomas and inflammatory processes, and can be used to monitor inflammatory processes (10, 11).

Globally, there has been an increasing number of studies demonstrating that several hematological inflammatory markers, including MPV, RDW, PDW, and NLR, can reflect the degree of inflammation in some acute and chronic diseases (8, 11, 12). To our knowledge, only one study used these parameters for the differential diagnosis of BEO and non-BEO (13). In the present study, we aimed to compare the levels of hematological inflammatory markers, including WBC, PLT, RDW, MPV, NLR, and PLR, between BEO and non-BEO. Additionally, we aimed to evaluate the utility of these parameters in the differential diagnosis of these two conditions.

## MATERIALS AND METHODS

We retrospectively reviewed 110 cases diagnosed with epididymo-orchitis who were followed up in our clinic between January 2012 and January 2017. Of these 110 patients, 38 were excluded due to malignancy, hematological problems, immunosuppressed state, cerebrovascular or coronary artery disease, or history of blood transfusion within the last month. Of the remaining 72 patients, 22 had BEO and 50 had non-BEO.

The diagnosis of epididymo-orchitis was based on a combination of symptoms (i.e., testicular pain, swelling, tenderness, and scrotal redness with no other identified cause) and laboratory results (i.e., complete blood count, C-reactive protein (CRP), and erythrocyte sedimentation rate (ESR)).

Diagnoses of BEO were made using positive clinical findings such as testicular pain, tenderness, and scrotal swelling, positive Rose Bengal test results, ≥ 1/160 standard tube agglutination (STA) titer values, and/or positive blood culture.

Blood samples were drawn via vacuum collection into tubes containing standard EDTA. All blood samples were analyzed within one hour after collection using a regularly calibrated analyzer (Abbott CELL-DYN Ruby Hematology System). Complete blood count parameters were recorded (i.e., WBC, neutrophil, monocyte, and PLT counts as well as RDW, PDW, PCT and MPV). NLR, PLR, and MLR were calculated as the ratio of neutrophils to lymphocytes, platelets to lymphocytes, and monocytes to lymphocytes, respectively. These parameters were compared between the BEO and non-BEO groups.

### Statistical analysis

The Kolmogorov Smirnov test was used to determine whether the distributions of continuous variables were normal. As applicable, descriptive statistics for continuous variables were expressed as mean±SD or median (25^th^-75^th^) percentiles. Mean differences between groups of normally distributed data were compared with Student's t test, while the Mann Whitney U test was used to compare non-normally distributed data.

Receiver operating characteristic (ROC) curves were constructed by calculating the sensitivities (true positive rate) and specificities (false positive rate) of each laboratory measurement. The diagnostic sensitivity, specificity, positive predictive value (PPV), and negative predictive value (NPV) were calculated according to the following formulas, where TP= true positive, TN = true negative, FP = false positive, and FN = false negative: Sensitivity = TP/(TP+FN), Specificity = TN/(TN+FP), Positive predictive value= TP/(TP+FP), and Negative predictive value= TN/(TN+FN).

A Multiple Logistic Regression Analysis with a Forward LR procedure was used to determine the best predictor(s) for discriminating between non-Brucella epididymo-orchitis and Brucella epididymo-orchitis. Any variable with p < 0.25 via univariate test was accepted as a candidate for the multivariate model, which also included all variables of known clinical importance. Odds ratios, 95% confidence intervals, and Wald statistics for each independent variable were also calculated. Data analysis was performed using IBM SPSS Statistics version 17.0 software (IBM Corporation, Armonk, NY, USA). Values of p less than 0.05 were considered significant.

## RESULTS

All BEO positive cases had positive Rose Bengal test results and ≥ 1/160 standard tube agglutination (STA) titer values. One of five patients (20%) with high fever and blood culture had positive blood culture for brucella species.

Table-1 compares the demographic properties and laboratory results of the BEO and non-BEO groups. The median MLR (p=0.002) and the median MPV, median WBC count, median neutrophil count, and median NLR were significantly lower in the BEO group (p < 0.001), while the median RDW and lymphocyte count were significantly higher (p < 0.001 and p=0.030, respectively). There were no significant differences between the groups regarding PLR or monocyte count (p > 0.05) (Table-1). In addition, NLR had a significant discriminative power in distinguishing between BEO and non-BEO (AUC=0.784, 95% CI: 0.670-0.897, p <0.001 (Table-2).

**Table 1:** Demographic and laboratory measurements of the two groups.

	Brucella	Non-brucella	p-value
	Epididymo-orchitis (n=22)	Epididymo-orchitis (n=50)	
Age	34.6±17.8	43.1±15.3	**0.043** †
WBC	8.5 (7.5-10.5)	12.6 (8.5-17.0)	**< 0.001** ‡
RDW	15.4 (13.0-17.2)	12.8 (11.6-14.1)	**< 0.001** ‡
Platelet count	266.3±56.3	264.3±92.1	0.923†
PDW	18.2 (17.6-19.8)	19.2 (18.1-20.4)	0.079‡
PCT	0.19 (0.14-0.23)	0.21 (0.16-0.24)	0.134‡
MPV	6.9 (6.5-7.5)	7.8 (7.1-9.7)	**< 0.001** ‡
PLR	108.2 (76.9-132.1)	113.1 (91.5-170.8)	0.136‡
Neutrophil	5.3 (3.5-7.3)	8.9 (5.9-12.8)	**< 0.001** ‡
Lymphocyte	2.59±0.87	2.12±0.82	**0.030** †
NLR	1.8 (1.2-4.0)	4.6 (3.0-7.7)	**< 0.001** ‡
Monocyte	0.78 (0.45-0.91)	0.81 (0.70-1.16)	0.052‡
MLR	0.28 (0.19-0.42)	0.48 (0.29-0.65)	**0.002** ‡

† = Student's t test, data shown as mean ± SD,

‡ = Mann Whitney U test, data expressed as median (25th-75th) percentiles.

**Table 2:** Results of ROC curve analyses.

	AUC	95% CI	p-value
WBC	0.744	0.632 - 0.856	**< 0.001**
RDW	0.737	0.602 - 0.873	**< 0.001**
Platelet count	0.540	0.405 - 0.675	0.591
PDW	0.630	0.496 - 0.765	0.079
PCT	0.611	0.472 - 0.751	0.134
MPV	0.738	0.623 - 0.853	**< 0.001**
PLR	0.611	0.472 - 0.750	0.136
Neutrophil	0.781	0.673 - 0.890	**< 0.001**
Lymphocyte	0.658	0.523 - 0.792	**0.034**
NLR	0.784	0.670 - 0.897	**< 0.001**
Monocyte	0.645	0.510 - 0.779	0.052
MLR	0.726	0.605 - 0.847	**0.002**

**AUC** = Area under the curve; **CI** = Confidence interval

The best cut-off value for discriminating between the BEO and non-BEO groups using MPV was 7.65 fL. According to ROC analysis, an MPV < 7.65 fL reduced the likelihood of BEO, while an MPV > 7.65 fL increased the likelihood of BEO. At this cut-off level, MPV had a sensitivity of 86.4%, a specificity of 62.0%, a positive predictive value of 50.0%, a negative predictive value of 91.2%, and a likelihood ratio of 69.5% (Table-3).

**Table 3:** Best cut-off points for laboratory measurements and diagnostic performance statistics.

	Cut-off point	Sensitivity	Specificity	PPV	NPV	Accuracy
WBC	< 12.95	95.5%	50.0%	45.7%	96.2%	63.9%
RDW	> 14.45	63.6%	82.0%	60.9%	83.7%	76.3%
MPV	< 7.65	86.4%	62.0%	50.0%	91.2%	69.5%
Neutrophil	< 7.895	90.9%	60.0%	50.0%	93.8%	69.5%
Lymphocyte	> 1.96	77.3%	54.0%	42.5%	84.4%	61.1%
NLR	< 2.3	59.1%	86.0%	65.0%	82.7%	77.8%
Monocyte	< 1.01	90.9%	38.0%	39.2%	90.5%	54.2%
MLR	< 0.45	81.8%	56.0%	45.0%	87.5%	63.9%

**PPV** = Positive predictive value; **NPV** = Negative predictive value

**Figure 1:**
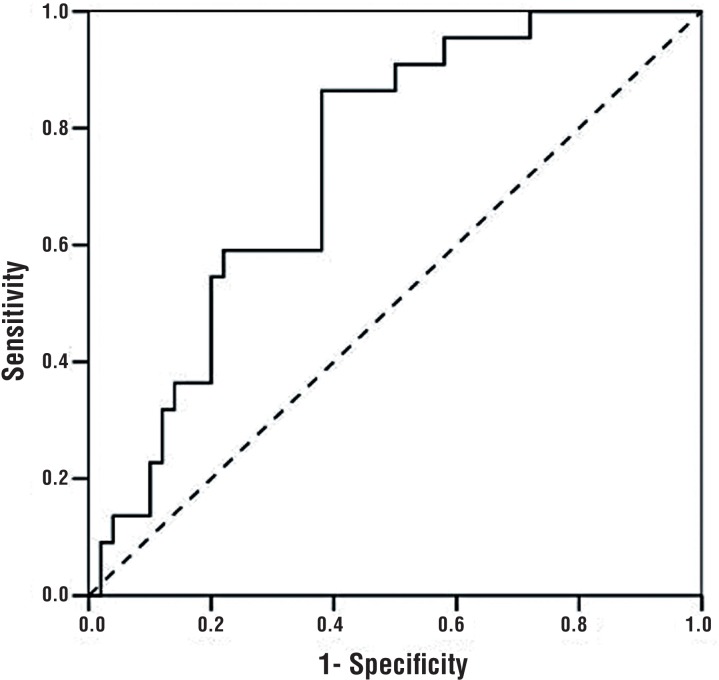
ROC curve for RDW.

**Figure 2:**
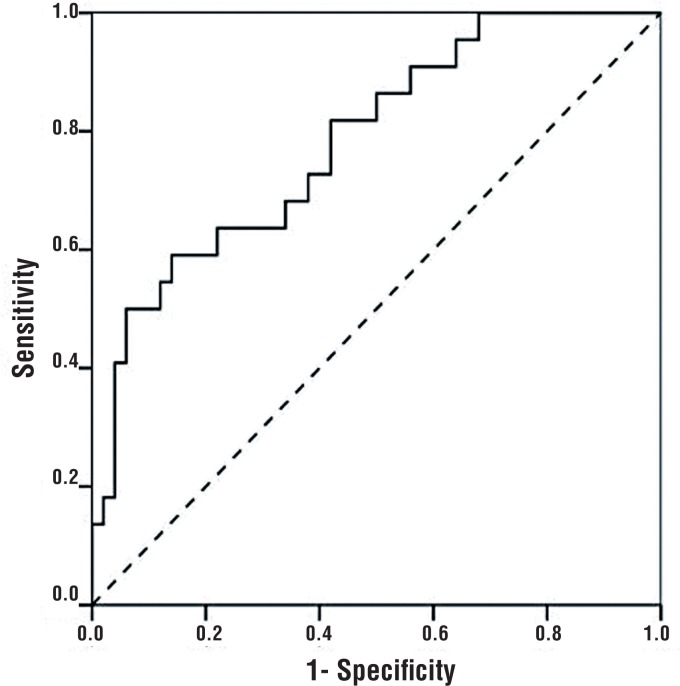
ROC curve for MPV.

**Figure 3:**
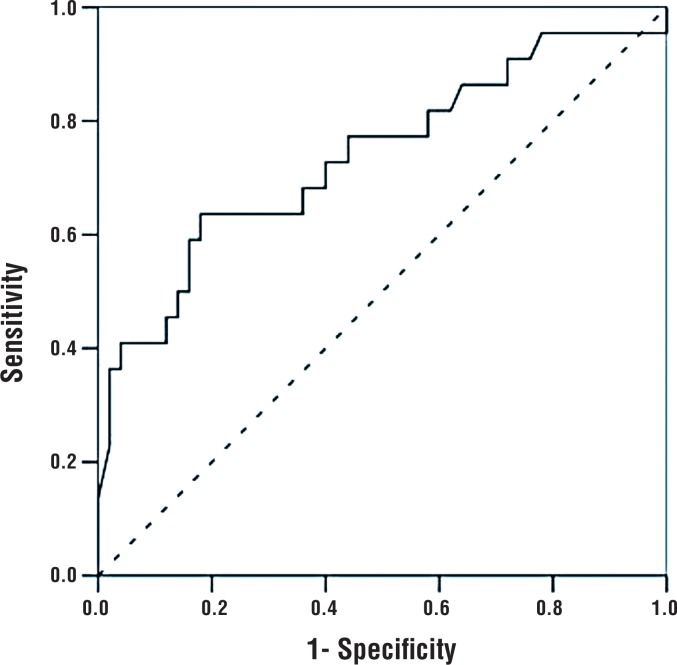
ROC curve for NLR.

**Figure 4:**
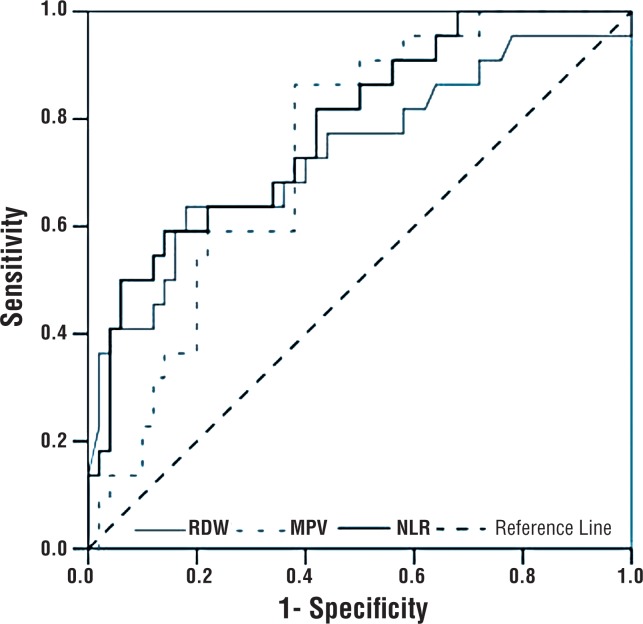
ROC curve for RDW, MPV, and NLR.

The most decisive factors for discriminating between BEO and non-BEO were NLR, RDW, and MPV, in decreasing order of strength. Regardless of other factors, an NLR value < 2.3 significantly increased the odds of BEO (OR=8.080, 95% CI: 1.929-33.843, p=0.004). After adjusting for other factors, an RDW value >14.45% significantly increased the odds of BEO (OR=7.020, 95% CI: 1.749-28.176, p=0.006). Independent of the other factors, patients with an MPV < 7.65 fL had a 6.336 times higher risk for BEO (95% CI: 1.393 -28.822, p=0.017) (Table-4) (Figures 1–4).

**Table 4:** Results of multiple logistic regression analyses.

	Odds ratio	95% Confidence interval		Wald	p-value
		Lower limit	Upper limit		
RDW > 14.45	7.020	1.749	28.176	7.554	**0.006**
MPV < 7.65	6.336	1.393	28.822	5.706	**0.017**
NLR < 2.3	8.080	1.929	33.843	8.175	**0.004**

## DISCUSSION

Brucellosis is one of the greatest imitators in the realm of infectious diseases, as it can mimic other multi-system diseases. It has wide clinical polymorphism, which can frequently lead to misdiagnosis and treatment delays. Humans can be infected with brucellosis by consumption of uncooked dairy products (as are consumed in southeastern parts of Turkey) and infected meat (goat, cattle, sheep, cow, camel and pork). Humans may also be infected via close contact with the secretions and carcasses of these animals. Clinically, the disease may have a subclinical, acute, subacute, or chronic course (4).

BEO is a common clinical complication of brucellosis. Its diagnosis is made upon the results of clinical, serological, and microbiological tests. In areas where the disease is endemic, laboratory facilities may not always be appropriate or adequate for diagnosing the disease. Since secondary antibody titers may remain high after treatment, it is difficult to determine whether treatment needs to be continued (14). For this reason, it is believed that supplementary diagnostic methods are beneficial throughout the diagnosis and follow-up period.

Non-specific laboratory findings have been reported for the diagnosis and differential diagnosis of BEO; these include leukocytosis, anemia, thrombocytopenia, elevated AST and ALT levels, and increased CRP and ESR (15-18,). Although some studies report a high incidence of leukocytosis (19, 20), this is not a typical feature of brucellosis (20, 21). Mild pancytopenia has also been reported in cases with BEO (21, 22). In our current study, we found that the BEO group had a significantly lower median WBC count than the non-BEO group. We did not detect pancytopenia in either of the groups. In addition, Aydın et al. reported that there was no significant difference between the mean WBC levels of BEO and non-BEO patients (13).

In brucellosis, acute phase reactants are elevated as a result of the inflammatory process (23). Indirect inflammatory markers have been defined, including MPV, PDW, RDW, NLR, and PLR.

MPV is a measure of platelet size, and is the most commonly used marker of platelet function. Modern blood counters calculate MPV during the routine complete blood count analysis, but this parameter is often overlooked by clinicians (24). MPV levels usually increase with mild and acute inflammation, and decrease in severe and chronic inflammation. MPV levels can reveal inflammatory burden and the presence of disease activity in many diseases (e.g., preeclampsia, acute pancreatitis, unstable angina, and myocardial infarction) and in cases of systemic inflammation (e.g., ulcerative colitis and Crohn's disease) (25).

Brucellosis is a systemic chronic inflammatory disease. Okan et al. found that cases with brucellosis had lower MPV levels than healthy controls (26), while Küçükbayrak et al. and Bozkurt et al. showed that these levels increased following treatment (27, 28). Aydın et al. compared the complete blood count parameters (WBC, PLT, RDW, MPV, NLR, PLR, and MLR) of BEO and non-BEO patients, and found significantly lower mean MPV in patients with BEO. To our knowledge, that study was the first of its kind published in English (13). In our current study, we found significantly lower median MPV in the BEO group. The best cut-off value for discriminating between BEO and non-BEO was 7.65 fL. Using this cut-off point, MPV showed a sensitivity of 86.4%, a specificity of 62.0%, a positive predictive value of 50.0%, a negative predictive value of 91.2%, and a likelihood ratio of 69.5%.

RDW is a measure of heterogeneity in the size of circulating red blood cells, and is commonly measured during standard complete blood count analyses. Several studies have reported altered RDW in various pathologies, including inflammatory bowel disease, celiac disease, pulmonary embolism, and coronary artery disease. Furthermore, its predictive value has been shown in various inflammatory and infectious pathologies, such as acute pancreatitis, bacteremia, sepsis, and septic shock (29). Elevated RDW is an expected finding in inflammatory and infectious pathologies, as reticulocytes are often released into the circulation during the early stages. In our current study, the median RDW level was significantly higher in the BEO group. After adjusting for other factors, an RDW level above 14.45% was associated with a significantly increased risk for BEO.

Neutrophils and lymphocytes play important roles in inflammatory processes. Neutrophil and lymphocyte counts show transient changes under inflammatory conditions (i.e., as neutrophil count increases, lymphocyte count decreases). NLR has been defined as a systemic inflammation index that is useful in the differential diagnosis or prognostic prediction of various diseases (30). However, changes in platelet/lymphocyte ratio (PLR) may be related to inflammation and cytokines. Similar to NLR, PLR is also used for the differential diagnosis and prognostic prediction of various diseases, including cancer and inflammatory conditions (31). Among studies investigating NLR and PLR in brucellosis, Olt et al. (32) reported that adult patients with brucellosis had significantly increased Hb and NLR, while Aktar et al. (31) reported that NLR and PLR were direct indicators of inflammation in children with Brucella-arthritis and Bozdemir et al. (34) found significantly altered Hb and NLR in children with brucellosis. Aydın et al. compared parameters including NLR, PLR, and MLR between patients with BEO and non-BEO, and found significantly higher MLR among patients with BEO; however, they found no differences between the two groups regarding NLR or PLR (13).

In our current study, patients with BEO had significantly lower median neutrophil count, median NLR, and median MLR, but higher lymphocyte count compared to the non-BEO group. However, there were no significant differences between the groups regarding PLR or monocyte count. Regardless of other factors, an NLR value lower than 2.3 was associated with a significantly increased risk for Brucella epididymo-orchitis.

There are some limitations to our study, including that it had a retrospective design and a relatively small sample size. There is a need for larger prospective studies to investigate the alterations in differential leukocyte count and platelet parameters with long-term follow-up in patients with BEO.

In conclusion, based on our current findings, we believe complementary hematological inflammatory markers (such as MPV, NLR, PLR, and RDW), which can be measured rapidly, easily, and with no additional cost, can be used in addition to the diagnostic serological tests to aid in the diagnosis, follow-up, and differential diagnosis of BEO. Further studies are needed to confirm these findings in a clinical setting, and to understand the underlying mechanisms for these findings.
